# Wedelolactone Inhibits Hepatitis B Virus Replication by Modulating NF-κB and Nrf2/HO-1 Signaling: An in-vitro Huh7 1.3-mer HBV Plasmid Model

**DOI:** 10.5812/ijpr-168329

**Published:** 2026-04-15

**Authors:** Bin Wang, Xuehui Bu, Umar Saeed, Zahra Zahid Piracha, Di Xiao

**Affiliations:** 1 Department of Hepatology, Public Health Clinical Center Affilliated to Shandong University, Jinan, China; 2 Clinical and Biomedical Research Center (CBRC), Foundation University School of Health Sciences (FUSH), Foundation University, Islamabad, Pakistan; 3 Korea University, Seoul, Republic of Korea; 4 Institute of Graduate Studies and Research, Cyprus International University, Nicosia, Northern Cyprus; 5 Széchenyi István University, Győr, Hungary

**Keywords:** Wedelolactone, Hepatitis B Virus, Antiviral Activity, HBV Replication, Inflammation, Oxidative Stress, NF-κB Signaling, Nrf2/HO-1 Pathway.

## Abstract

**Background:**

Chronic hepatitis B virus (HBV) infection is a well-recognized cause of hepatic injury through prolonged viral replication, inflammation, and oxidative stress. Existing antiviral drugs limit viral replication but cannot eliminate viral transcription or even totally preclude liver injury, thus reemphasizing the significance of drugs with combined antiviral and hepatoprotective effects.

**Objectives:**

To evaluate the effects of wedelolactone on HBV replication, gene expression, inflammation, and oxidative stress in an in-vitro model of HBV plasmid transfection with human hepatic cells.

**Methods:**

Human hepatocellular carcinoma cells (Huh7) were transfected with a 1.3-mer plasmid and treated with wedelolactone (2.5 - 10 µM). Luciferase assays for HBV promoter activity, Northern blotting and Southern blotting for transcripts and replicative intermediates, qPCR for extracellular HBV DNA, and western blotting for viral antigens such as HBx were performed. Cell cytotoxicity was measured. NF-κB/IκB, inflammatory cytokines (TNF-α, IL-6), and antioxidant markers (Nrf2, HO-1, Keap1) were assessed to evaluate inflammatory and oxidative responses.

**Results:**

Wedelolactone significantly suppresses HBV promoter activity, RNAs, core particle formation, and extracellular HBV DNA. It reduced the expression of HBcAg and HBsAg. It inhibited NF-κB activation and cytokine release, while simultaneously enhancing Nrf2/HO-1 signaling, including induction of heme oxygenase-1 by lowering levels of Keap1.

**Conclusions:**

Wedelolactone exerts dual antiviral and hepatoprotective actions by inhibiting HBV replication and modulating inflammatory and oxidative stress pathways.

## 1. Background

Hepatitis B virus (HBV) infection remains a major global health burden, with more than 250 million people chronically infected and at risk for progressive liver disease, including cirrhosis and hepatocellular carcinoma (1, 2). Although effective vaccines and antiviral agents are available, including nucleos(t)ide analogues, current therapies rarely achieve a functional cure, in large part because such therapies fail to eradicate covalently closed circular DNA or completely suppress viral transcription (3, 4). Prolonged nucleos(t)ide analogue (NA) therapy often results in drug resistance and does not improve HBV-induced hepatic inflammation and oxidative stress, which have been implicated as central to liver damage and carcinogenesis (5, 6). These limitations make the identification of novel antiviral agents capable of suppressing HBV replication and mitigating liver injury an urgent need. Natural products have been recognized as an infinitely large source of antiviral and hepatoprotective agents (7, 8). Wedelolactone, a coupled compound derived from the plant Eclipta prostrata, has been used as part of liver tonics and has been ascribed various biological activities, including its anti-inflammatory, antioxidant, and anticancer effects (9, 10). Research has shown that wedelolactone activates many important biological pathways, including NF-κB and nuclear factor erythroid 2-related factor 2 (Nrf2), both important pathways involved in the pathogenesis of HBV and liver damage (11-13). However, its capability to play a role in controlling the transcriptional activity, viral protein expression, or replication of HBV has never been evaluated. Additionally, its ability to counteract the resulting oxidative damage and inflammation caused during HBV replication is also unknown. To date, few studies have integrated antiviral and hepatoprotective modes in a systematic, mechanism-based approach using in-vitro HBV models. Most published works lack molecular evidence, or the suppression of viral replication is not related to downstream immune modulation or redox balance (14). This is an important knowledge gap, especially for the development of multifunctional agents to eventually complement current antiviral regimens or serve as templates in drug discovery.

## 2. Objectives

Antiviral and cytoprotective activities of wedelolactone were investigated in this study, focusing on HBV-infected hepatocytes. To this end, a plasmid-based HBV replication system in hepatocellular carcinoma cells (Huh7) hepatoma cells was employed. We evaluated the antiviral activities of wedelolactone on HBV promoter activities, RNA transcription, core particle formation, and extracellular viral DNA. Furthermore, we extended this investigation to the assessment of wedelolactone’s potential in mitigating inflammatory and oxidative stress pathways, involving NF-κB and cytokine production, and the Nrf2/HO-1 signaling pathway. This investigation clearly underscores the potential of wedelolactone as a promising lead compound for adjunctive treatment in the clinical management of hepatitis B.

## 3. Methods

### 3.1. Cell Culture and Reagents

hepatitis B virus hepatoma cells (ATCC, USA) were cultured in Dulbecco’s Modified Eagle Medium (DMEM; Gibco) supplemented with 10% fetal bovine serum (FBS), 100 U/mL penicillin, and 100 µg/mL streptomycin at 37°C in a 5% CO₂ incubator. Wedelolactone (Sigma-Aldrich) was dissolved in DMSO to prepare a 10 mM stock and diluted to working concentrations of 2.5, 5, and 10 µM in culture medium. Final DMSO content did not exceed 0.1% (14).

### 3.2. HBV Transfection

Hepatitis B virus replication was established using a 1.3-mer HBV genome plasmid. Cells were seeded in six-well plates and transfected with 2 µg of HBV plasmid using Lipofectamine 3000 (Invitrogen) following the manufacturer’s instructions. Mock-transfected cells served as negative controls. Cells were treated with wedelolactone 6 h post-transfection and harvested 48 h later for downstream analyses.

### 3.3. Cell Viability Assay

The MTT assay was used to assess the cytotoxicity of wedelolactone. The wedelolactone concentration range (2.5 - 10 µM) was selected based on prior studies reporting biologically active, low-micromolar dosing of wedelolactone in hepatic inflammation and oxidative stress models, and was capped at 10 µM to remain within a conservative, non-cytotoxic exposure window for hepatocyte-derived cells (11-13). Following 48 h treatment, cells were incubated with 20 µL of 5 mg/mL MTT (Sigma-Aldrich) for 4 h. The resulting formazan crystals were solubilized in DMSO, and absorbance was recorded at 570 nm using a microplate reader (BioTek, USA) (15).

### 3.4. Luciferase Reporter Assay

Hepatocellular carcinoma cells were transfected with firefly luciferase reporter constructs driven by the HBV EnhII/Core (EnhII-Cp), EnhI/X (EnhI-Xp), PreS1, or PreS2 promoters. Following transfection, cells were treated with wedelolactone (2.5, 5.0, and 10.0 µM) or vehicle (HBV-only; 0 µM) for the same duration used in the experiment. Cells were then lysed, and lysates were mixed with luciferase assay reagent according to the manufacturer’s instructions. Firefly luminescence (relative light units; RLU) was measured using a luminometer/illuminator. To control for inter-assay variability, promoter activity at each dose was expressed relative to the corresponding HBV-only (0 µM) control, which was set to 100%: Normalized activity (%) = (RLU_dose / mean RLU_HBV-only) × 100. Percent inhibition at 10 µM was calculated as: Inhibition (%) = 100 − normalized activity (% at 10 µM). Data are presented as mean ± SD (n = 3). Statistical significance was assessed by one-way ANOVA followed by Tukey’s post hoc test.

### 3.5. RNA Extraction and Northern Blotting

Total RNA was extracted using TRIzol reagent (Invitrogen), and RNA quantity was determined spectrophotometrically. For Northern blotting, 20 µg of RNA was denatured at 65 °C, electrophoresed on 1.2% formaldehyde-agarose gels (Invitrogen), and transferred to nylon membranes (Roche). Membranes were hybridized at 68°C for 4 h to a 32P-labeled random-primed probe specific for the full-length HBV sequence.

### 3.6. Hepatitis B Virus DNA Detection by Southern Blot and qPCR

Intracellular HBV DNA was isolated from core particles using a detergent lysis and DNase digestion protocol. DNA samples were resolved on agarose gels, transferred to nylon membranes (Whatman), and hybridized with a 32P-labeled random-primed probe specific for full-length HBV. Extracellular HBV DNA was extracted from culture supernatants using the QIAamp MinElute Virus Spin Kit (Qiagen) and quantified via qPCR with HBV-specific primers.

### 3.7. Western Blotting

Protein lysates were prepared using RIPA buffer supplemented with protease and phosphatase inhibitors (Roche). Equal amounts of protein (30 µg) were resolved by SDS-PAGE and transferred to PVDF membranes (Millipore). Membranes were blocked with 5% non-fat milk and probed with primary antibodies against cleaved caspase-3, cleaved PARP, Bcl-2, HBcAg, HBsAg, NF-κB p65, IκBα, Nrf2, HO-1, Keap1, and GAPDH (loading control). After incubation with HRP-conjugated secondary antibodies, signals were visualized using ECL (Thermo).

### 3.8. Core Particle Immunoblotting

Four percent of the total lysate was electrophoresed in 1% native agarose gels, and resolved core particles were transferred to PVDF membranes. Immunoblotting to visualize core particles was performed using a polyclonal rabbit anti-HBc primary antibody (1:1,000 dilution) (generated in-house), followed by a horseradish peroxidase-conjugated anti-rabbit secondary antibody (1:5,000 dilution) (Thermo Fisher Scientific) (16).

### 3.9. HBsAg Quantification by ELISA

Secreted HBsAg levels were determined in culture supernatants using a commercial HBsAg ELISA kit (BioVendor), following the manufacturer's protocol. Absorbance was read at 450 nm.

### 3.10. qPCR for Inflammatory and Antioxidant Genes

Expression levels of inflammatory (TNF-α, IL-6) and antioxidant (Nrf2, HO-1, NQO1) genes were assessed by real-time PCR using cDNA prepared from TRIzol-isolated RNA. GAPDH was used as the internal control, and relative expression levels were determined using the 2^-ΔΔCt^ method. Primer specificity was confirmed by melt-curve analysis and no-template controls, in accordance with key MIQE reporting recommendations.

### 3.11. Statistical Analysis

All experiments were performed as three independent biological replicates and are presented as mean ± SD. For experiments including mock, HBV-only (vehicle), and wedelolactone dose groups, overall differences were assessed by one-way ANOVA. The prespecified primary comparisons were each wedelolactone dose versus the HBV-only (vehicle) group; mock was included to define baseline and confirm HBV-induced changes. Tukey’s post-hoc test was applied to control for multiple comparisons. P < 0.05 was considered statistically significant.

## 4. Results

### 4.1. Wedelolactone Maintains Cell Viability and Does Not Induce Apoptosis in HBV-Infected Hepatocytes

**Figure 1.**
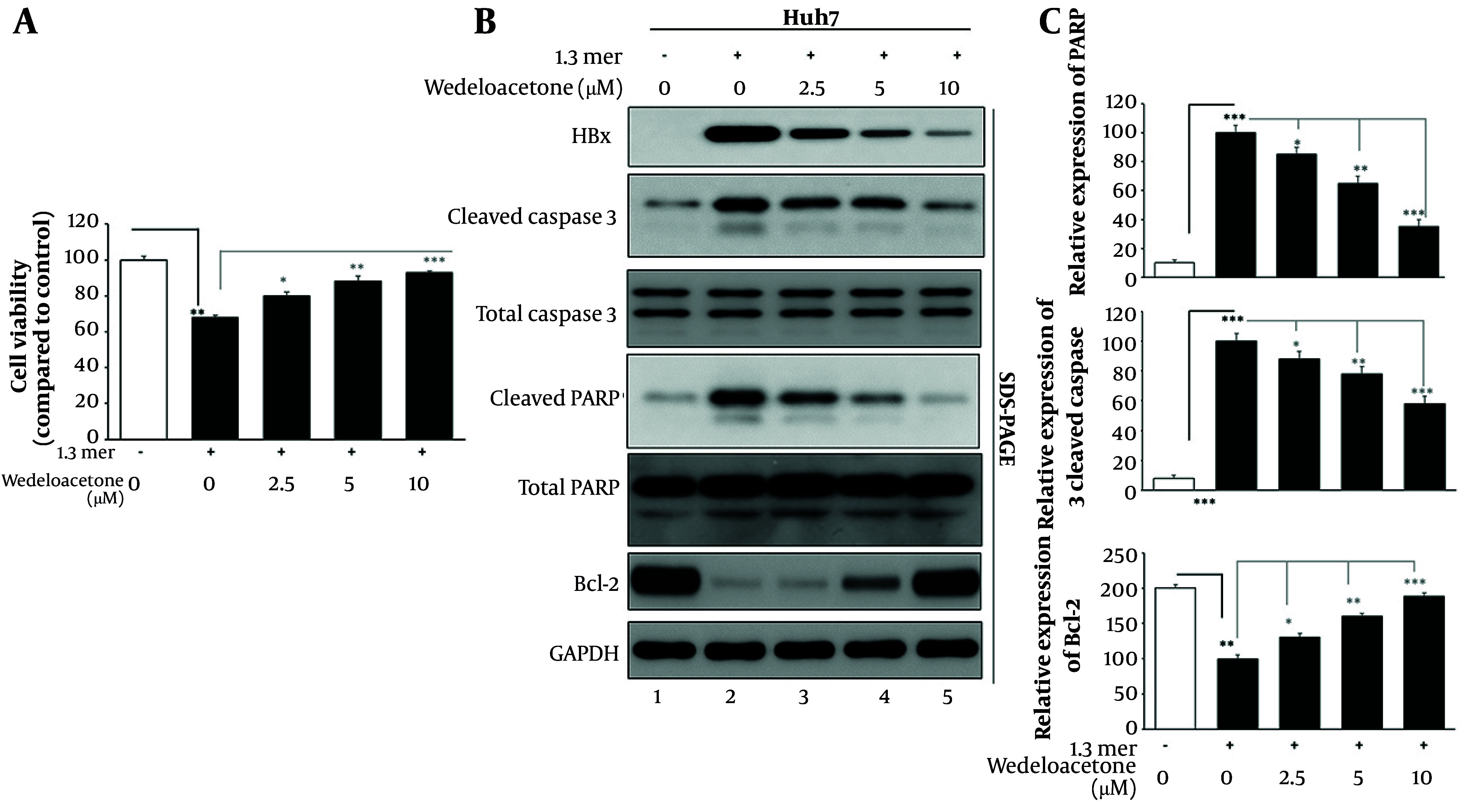
Evaluation of cytotoxicity and apoptosis modulation by wedelolactone in hepatitis B virus (HBV)-infected hepatocytes. A, hepatocellular carcinoma cells (Huh7) transfected with 1.3-mer HBV plasmid were treated with increasing concentrations of wedelolactone (2.5, 5, and 10 µM) for 48 hours. Cell viability was assessed using the MTT assay. B, Western blotting was performed to detect cleaved caspase-3, cleaved PARP, Bcl-2, and HBx expression in whole-cell lysates from mock, HBV-only, and wedelolactone-treated groups. C, Densitometric analysis of protein bands was conducted using ImageJ. Cleaved caspase-3 levels were normalized to total caspase-3, cleaved PARP was normalized to total PARP, and Bcl-2 was normalized to GAPDH. All experiments were performed in triplicate, and data are presented as mean ± SD. Statistical significance was determined using one-way ANOVA with Tukey’s post-hoc test; P < 0.05 was considered significant.

**Figure 2.**
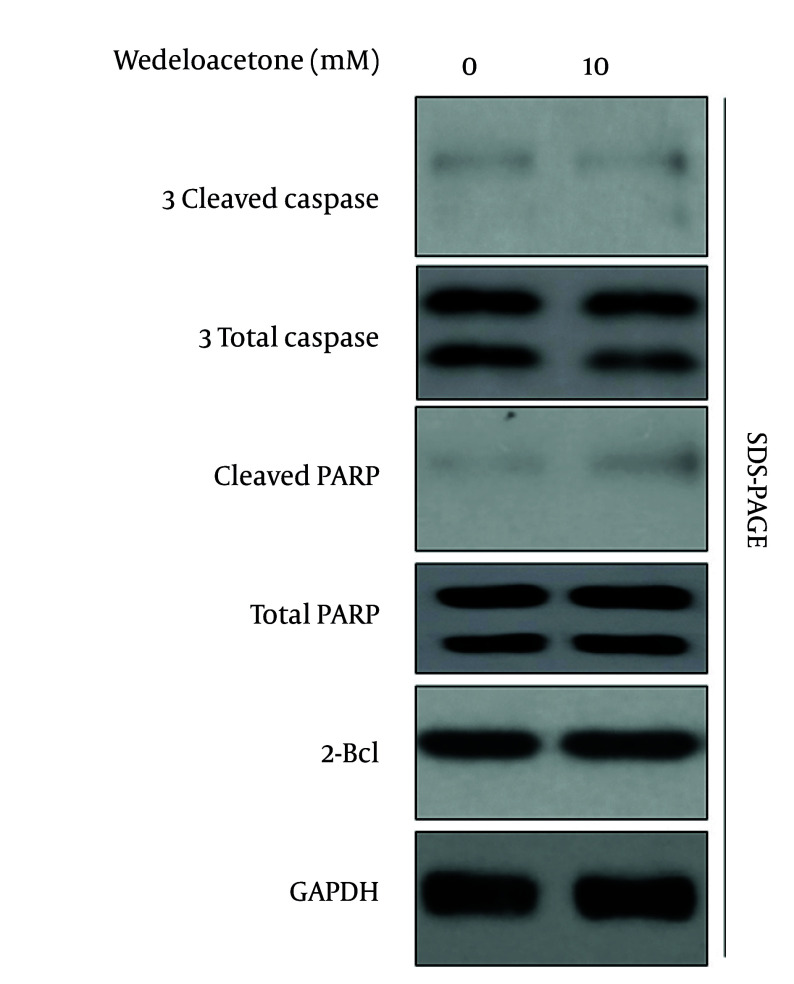
Wedelolactone does not alter baseline apoptosis signaling in mock-transfected hepatocellular carcinoma cells (Huh7). Mock-transfected Huh7 cells were treated with vehicle or wedelolactone (10 µM) for 48 h under the same exposure conditions as Figure 1. Whole-cell lysates were analyzed by Western blotting for Bcl-2, cleaved caspase-3, and cleaved PARP, with GAPDH used as a loading control.

To assess the cytotoxic potential of wedelolactone in the context of HBV replication, Huh7 containing 1.3-mer HBV were exposed to escalating doses of wedelolactone (2.5, 5.0, and 10.0 µM) for 48 hours, and viability was examined by a cell proliferation assay (MTT) (Figure 1A). The viability of 1.3-mer HBV transfection relative to mock cells (100%) was significantly decreased (68%) (P < 0.01). The addition of escalating doses of wedelolactone significantly improved viability in a concentration-dependent fashion, which upregulated viability at 2.5, 5.0, and 10.0 µM concentrations to 80% (P < 0.05 vs. 1.3-mer HBV-transfected cells), 88% (P < 0.01 vs. 1.3-mer HBV-transfected cells), and 93% (P < 0.001 vs. 1.3-mer HBV-transfected cells), respectively. These studies show that wedelolactone is a non-cytotoxic agent in this concentration range and also partially reactivates HBV-infected cells, as shown by a significant increase at the 10.0 µM concentration. To further confirm the apoptotic status, the expression of cleaved caspase-3, cleaved PARP, and Bcl-2 was analyzed by western blot assay (Figure 1B and C). As shown in Figure 1B, in addition to the expression of 1.3-mer, HBV infection significantly induced the expression of cleaved caspase-3 in 1.3-mer-transfected Huh7 compared to mock cells from 8.0 % to 100.0 % (P < 0.001, n = 3). Moreover, cleaved PARP expression was also significantly induced by HBV infection from 10 percent in mock cells to 100.0 percent in HBV-treated cells (P < 0.001, n = 3). Specifically, in HBV-treated cells, cleaved caspase-3 expression was significantly inhibited by wedelolactone to 88 percent, 78 percent, and 58 percent at 2.5, 5, and 10 μM wedelolactone, respectively (~12 percent, ~22 percent, ~42 percent reduction compared to HBV-only-treated cells; P < 0.05, P < 0.01, P < 0.001 vs. HBV-treated cells, by one-way ANOVA test and Tukey’s comparison test). Similarly, the decrease in the level of cleaved PARP also reached a nadir of 85.0 %, 65.0 %, and 35.0 % (~15%, 35%, and 65% reduction; P < 0.05, P < 0.01, and P < 0.001 vs. HBV). Finally, the expression of Bcl-2, which is reduced from 200.0 % in mock cells to 100.0 % in HBV-infected cells (P < 0.01), is significantly increased and even overexpressed with wedelolactone treatment, at a level of 130.0 %, 160.0 %, and 190.0 % of the level in HBV-infected cells at wedelolactone treatment concentrations of 2.5, 5, and 10 µM (~1.3-, 1.6-, and 1.9-fold; P < 0.05, P < 0.01, and P < 0.001 vs. HBV-infected cells). To clarify whether wedelolactone non-specifically modulates apoptosis-related signaling under basal conditions, we performed the same apoptosis panel in mock-transfected cells treated with vehicle or wedelolactone under identical exposure conditions. In mock-transfected cells, wedelolactone did not produce a pro-survival shift, showing no meaningful increase in Bcl-2 above baseline and no suppression of basal cleaved caspase-3 or cleaved PARP (Figure 2). These findings support that the anti-apoptotic changes observed in HBV-transfected cells primarily reflect mitigation of HBV-associated stress rather than a generalized anti-apoptotic effect. HBx protein levels also exhibited a clear dose-dependent decrease in response to wedelolactone (lanes 3 - 5), further supporting its role in mitigating HBV-induced apoptotic and pathogenic signaling. Taken together with the preserved cell viability in the MTT assay (Figure 1A), these findings indicate that wedelolactone is non-cytotoxic to HBV-infected hepatocytes and may protect against HBV-induced apoptotic cell death, thereby establishing a safe and biologically favorable profile for subsequent antiviral and hepatoprotective investigations.

### 4.2. Wedelolactone Suppresses Hepatitis B Virus Promoter Activity in a Dose-Dependent Manner

**Figure 3.**
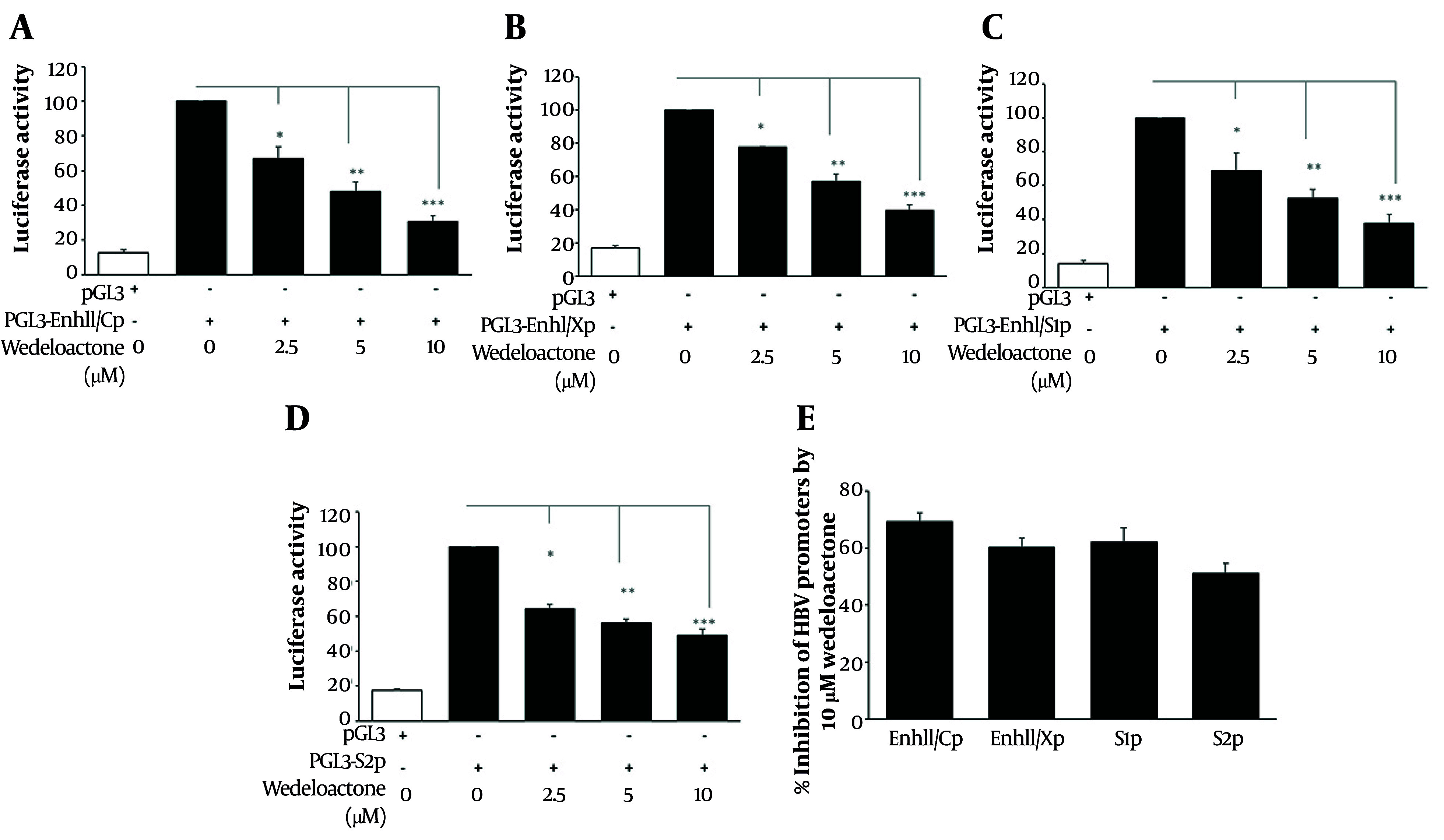
Analysis of hepatitis B virus (HBV) promoter activity in response to wedelolactone. A-D, hepatocellular carcinoma cells (Huh7) were transfected with HBV promoter-driven firefly luciferase reporter plasmids (EnhII/Cp, EnhI/Xp, PreS1, PreS2) cloned in the pGL3-basic vector. Cells were treated with wedelolactone (2.5, 5.0, and 10.0 µM) for 48 h post-transfection, lysed, and firefly luciferase activity (RLU) was measured using a luminometer. Luciferase signals at each dose were normalized to the corresponding HBV-only (0 µM/vehicle) control, which was set to 100%. The pGL3-basic empty vector was used as a negative control to determine background activity. E, percent inhibition at 10 µM wedelolactone was calculated as 100 − normalized activity (% at 10 µM). All experiments were performed in triplicate (n = 3), and data are presented as mean ± SD. Statistical significance was determined using one-way ANOVA with Tukey’s post hoc test; P < 0.05 was considered significant.

To determine the effect of wedelolactone on HBV promoter transcriptional activity, the effect on each individual promoter was examined using luciferase reporters (Figure 3). hepatocellular carcinoma cells were transiently transfected with firefly luciferase vectors driven by Enhancer II/Core (Enh2-CP), Enhancer I/X (Enh1-XP), PreS1, or PreS2 promoters and treated with wedelolactone (2.5, 5.0, or 10.0 µM) for 48 h. Basal amounts from the empty vector pGL3 were found to be lower (≈12 to 18 percent, as opposed to signals from each individual promoter) and were not impacted by wedelolactone, demonstrating its specificity for the target genes involved. Wedelolactone markedly suppressed Enh2-CP–driven transcription (Figure 3A). Relative to Enh2-CP (100.0 %), promoter activity decreased to 67.2 %, 48.1 %, and 30.7 % at 2.5, 5, and 10 µM, respectively (P < 0.05, P < 0.01, and P < 0.001 vs. 0 µM, one-way ANOVA with Tukey’s post hoc test; n = 3). A similar pattern was observed for Enh1-XP (Figure 3B), where wedelolactone reduced activity from 100.0 % to 77.9 %, 57.3 %, and 39.6 % at 2.5, 5, and 10 µM, respectively (P < 0.05, P < 0.01, and P < 0.001). PreS1 promoter activity (Figure 3C) was likewise inhibited, falling from 100.0 % to 69.0 %, 52.4 %, and 38.0 % across the same concentration range (P < 0.05 for 2.5 µM; P < 0.01 for 5 and 10 µM). PreS2 promoter activity (Figure 3D) also declined in a graded fashion, from 100.0 % to 64.6 %, 56.3 %, and 49.0 % at 2.5, 5, and 10 µM, respectively (P < 0.05 for 2.5 and 5 µM; P < 0.01 for 10 µM). Collectively, these data demonstrate that wedelolactone exerts a consistent, dose-dependent repression of all major HBV regulatory elements—Enh2-CP, Enh1-XP, PreS1, and PreS2 — without altering basal vector activity, supporting a specific inhibitory effect on HBV transcriptional control. Next, we quantified the percent inhibition of promoter activity at 10 µM wedelolactone relative to untreated controls, as calculated in the Methods (Figure 3E). EnhII–Cp showed the strongest repression, with activity inhibited by 69.3 %. EnhI–Xp and PreS1 were inhibited by 60.4 % and 62.0 %, respectively, while PreS2 showed a more moderate but still significant inhibition of 51.0 % (all P < 0.01 vs. 0 µM, one-way ANOVA with Tukey’s post hoc test). These data indicate that wedelolactone broadly targets HBV transcriptional regulation by suppressing multiple viral promoter regions, supporting the idea that its downstream effects on HBV RNA synthesis and replication arise, at least in part, from transcriptional repression at the promoter level.

### 4.3. Wedelolactone Suppresses Hepatitis B Virus RNA Transcription in Infected Hepatocytes

**Figure 4.**
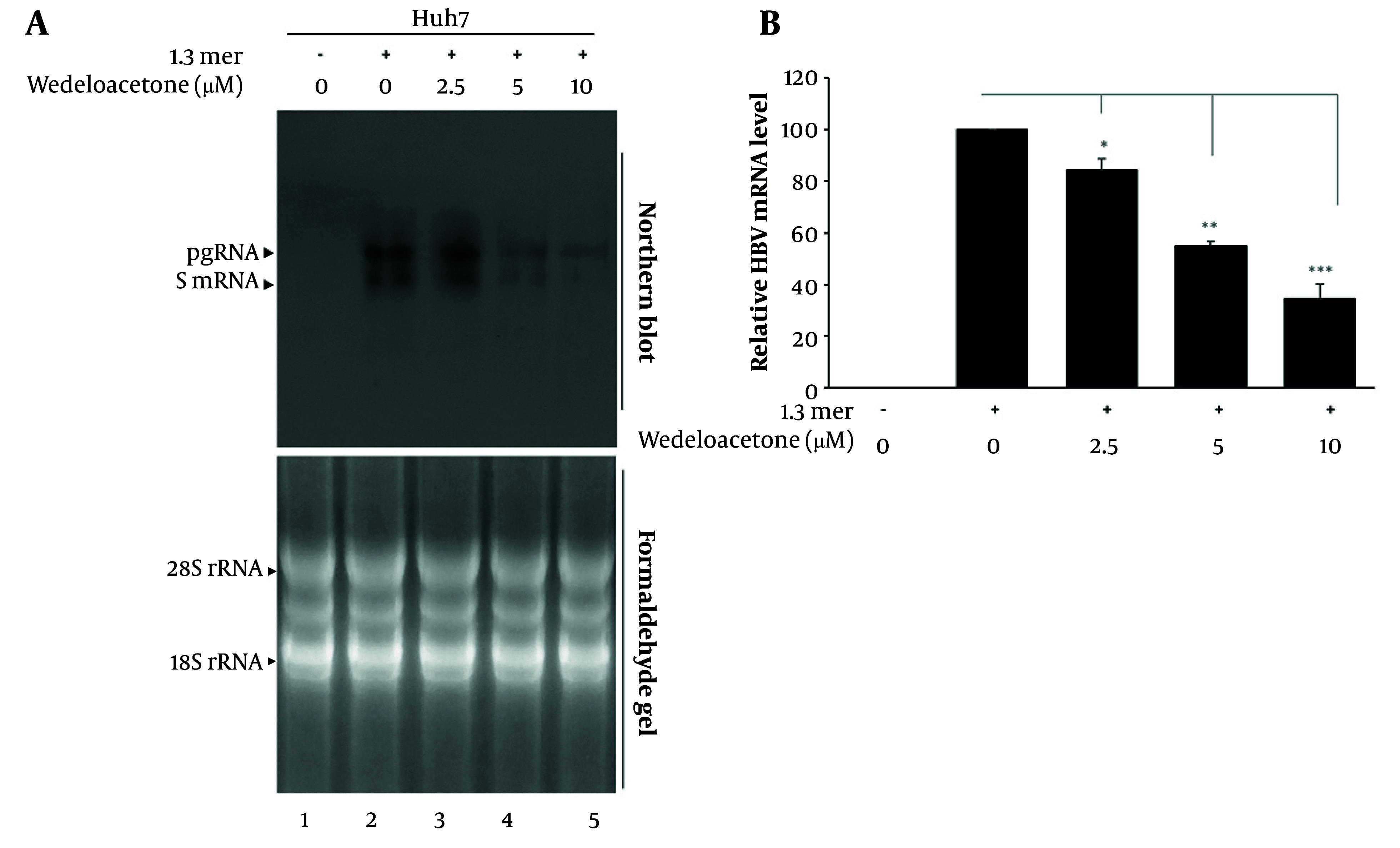
Wedelolactone inhibits hepatitis B virus (HBV) RNA transcription in hepatocellular carcinoma cells (Huh7). A, total RNA (20 µg per sample) was extracted from mock-transfected, HBV-only, and wedelolactone-treated Huh7 cells (2.5, 5, and 10 µM for 48 hours), separated on 1% formaldehyde-agarose denaturing gels, and transferred to nylon membranes. Membranes were hybridized with a random-primed 32P-labeled full-length HBV-specific DNA probe and visualized by autoradiography. The 3.5-kb pregenomic RNA (pgRNA) and 2.4/2.1-kb mRNAs encoding HBV surface proteins are indicated. Ethidium bromide-stained 28S/18S rRNA bands were used as RNA loading controls. B, densitometric analysis of autoradiographic signals was performed using ImageJ software. Band intensities were expressed as percentages relative to the HBV-only group. Results are presented as mean ± SD from three independent experiments. A P-value < 0.05 was considered statistically significant.

To assess the impact of wedelolactone on viral RNA synthesis, we evaluated the abundance of HBV transcripts following treatment using Northern blotting (Figure 4). Our data show that Northern blot analysis detected HBV 3.5 kb pregenomic RNA (pgRNA) and 2.4/2.1 kb S transcripts in five groups: Mock-transfected cells, HBV-only (1.3-mer), and HBV plus wedelolactone at 2.5 µM, 5.0 µM, and 10.0 µM (Figure 4A). In HBV-infected cells, both pgRNA and S RNA were strongly expressed (lane 1 vs. 2). Wedelolactone treatment led to a concentration-dependent reduction in the intensity of all HBV RNA species, with the greatest decrease observed at 10 µM (lanes 3-5). The 28S/18S rRNA formaldehyde gel ensures equal RNA loading across samples. As shown in Figure 4B, densitometry analysis showed that wedelolactone induced a clear, dose-dependent decrease in HBV RNA levels. Compared with 1.3-mer HBV cells set at 100%, HBV RNA declined to 84% at 2.5 µM, 55% at 5 µM, and 35% at 10 µM, corresponding to an overall reduction of about 65% at the highest dose. These findings indicate that wedelolactone markedly suppresses HBV RNA expression, likely as a downstream consequence of its inhibitory effects on viral promoter activity.

### 4.4. Wedelolactone Inhibits Hepatitis B Virus Replication and Viral Antigen Expression

**Figure 5.**
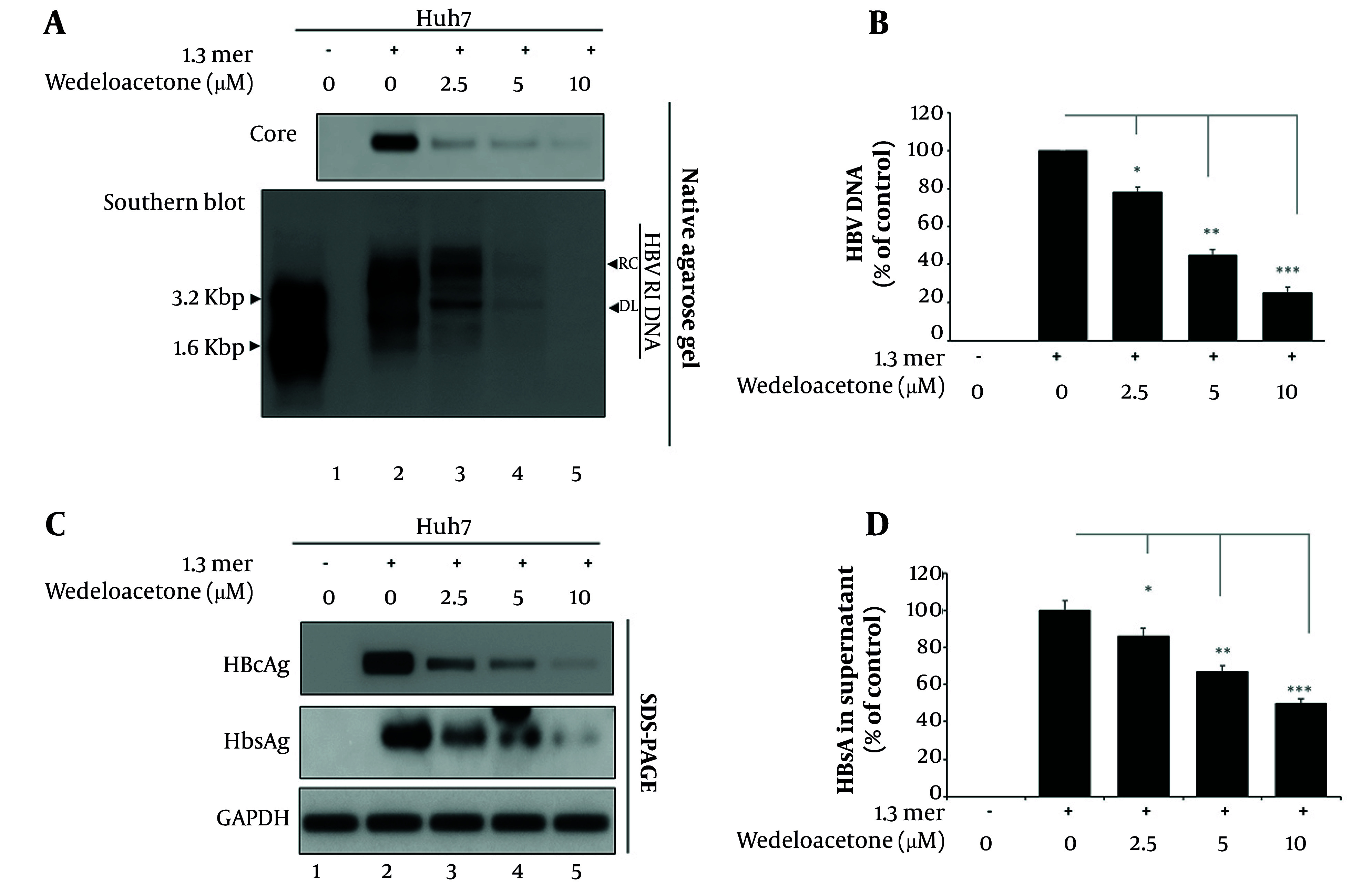
Suppression of hepatitis B virus (HBV) replication and viral antigen expression by wedelolactone. A, core particles were isolated from mock, HBV-only, and wedelolactone-treated hepatocellular carcinoma cells (Huh7) (2.5, 5, and 10 µM for 48 hours). Core particle-associated HBV capsids were detected by immunoblotting using an anti-HBc antibody across five sample groups. Core particle-associated HBV DNA was extracted from transfected Huh7 cells and subjected to Southern blotting to detect replicative intermediates (RC and DL forms). B, extracellular HBV DNA in culture supernatants was quantified by densitometry analysis. C, Western blotting was performed to detect intracellular HBcAg and HBsAg protein levels. D, secreted HBsAg levels in culture medium were measured using a commercial ELISA kit. Quantitative data are expressed as mean ± SD from three independent experiments. Statistical analysis was conducted using one-way ANOVA; P < 0.05 was considered significant.

To further evaluate the antiviral potential of wedelolactone, we assessed its effects on HBV replication and viral protein expression using core particle DNA analysis, extracellular DNA quantification, and protein detection assays (Figure 5). Core particle formation was robust in Huh7 cells transfected with the 1.3-mer HBV plasmid but was markedly reduced with increasing concentrations of wedelolactone (lanes 3 - 5). We first explored intracellular HBV DNA synthesis using Southern blot assays (Figure 5A). Replicative intermediates including relaxed circular and double-stranded linear DNAs were readily detectable in these 1.3-mer-transfected Huh7 cells (lane 2 vs. lane 1). A dose-dependent decrease in these intermediate molecules was noted upon treatment with wedelolactone (10 µM). Consistently, HBV DNA levels quantified by densitometry (Figure 5B) showed a significant, dose-dependent decline. Relative to HBV-only cells (100 %), HBV DNA was reduced to 78 % at 2.5 µM (22% decrease, P < 0.05), 45 % at 5 µM (55% decrease, P < 0.01), and 25 % at 10 µM (75% decrease, P < 0.001). These data indicate that wedelolactone effectively suppresses HBV replication at the level of both intracellular replicative intermediates and secreted viral DNA. In order to examine whether there was an effect on viral protein synthesis among infected cells, a western blot for HBcAg and HBsAg was carried out (Figure 5C). Results indicated that both of these viral antigens were highly expressed in infected cells (lane 2) but became significantly reduced in expression following wedelolactone treatment at each concentration from 2.5 µM to 10 µM (lanes 3 - 5). Quantitative measurement of secreted HBsAg in wedelolactone-treated culture supernatant was also performed through ELISA (Figure 5D), revealing a dose-dependent reduction relative to cell culture infection with 1.3-mer HBV (100%), with wedelolactone concentrations of 2.5 µM yielding 86% (14% reduction) of infection (~0.86-fold of control) (P < 0.05), 5 µM yielding 67% (33% reduction) (~0.67-fold of control) (P < 0.01), and 10 µM yielding 50% (50% reduction) (~0.5-fold of control) (P < 0.001) with regard to suppression of HBV particles in culture supernatant. Collectively, these findings demonstrate that wedelolactone effectively suppresses multiple stages of the HBV life cycle, including intracellular DNA replication, viral protein expression, and extracellular virion release.

### 4.5. Wedelolactone Modulates Inflammation and Oxidative Stress Signaling Pathways in hepatitis B virus-Infected Hepatocytes

**Figure 6.**
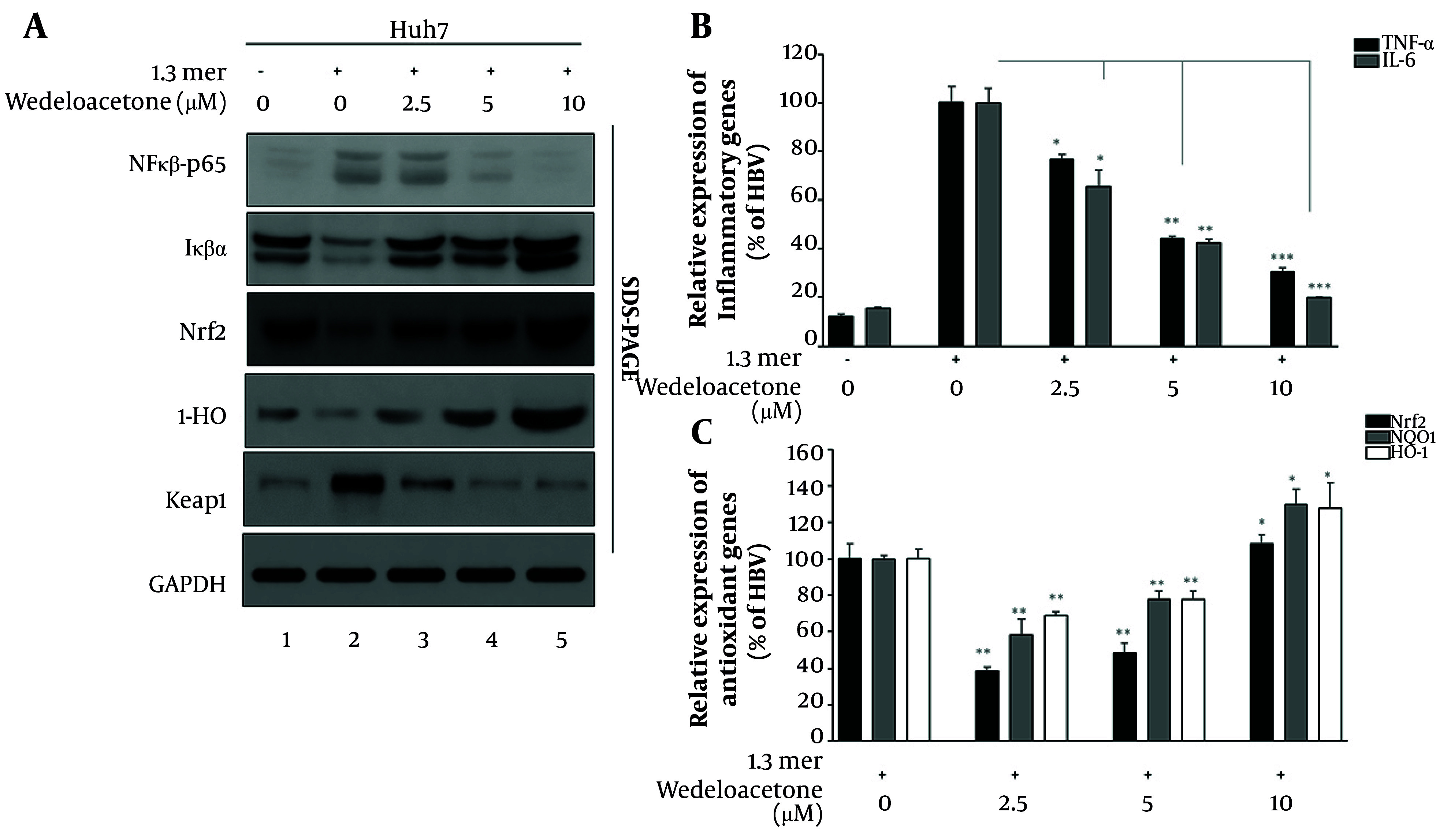
Modulation of inflammatory and oxidative stress pathways by wedelolactone. A, Western blotting was used to detect NF-κB p65, IκBα, Nrf2, HO-1, and Keap1 in cell lysates from mock and hepatitis B virus (HBV)-infected hepatocellular carcinoma cells (Huh7) treated with wedelolactone (2.5, 5, and 10 µM for 48 h). B, real-time PCR analysis was performed for proinflammatory cytokines TNF-α and IL-6. C, real-time PCR was also conducted to measure expression of antioxidant genes Nrf2, HO-1, and NQO1. All gene expression data were normalized to GAPDH. All qPCR and densitometric data are shown as mean ± SD from three replicates. Significance was assessed via one-way ANOVA with post-hoc correction; P < 0.05 was considered significant.

To delineate the molecular basis of wedelolactone’s hepatoprotective effects, we assessed the expression of key proteins and genes involved in inflammatory and oxidative stress pathways (Figure 6). Figure 6A shows western blot analysis of NF-κB p65, IκBα, Nrf2, HO-1, and Keap1 in HBV-infected Huh7 cells treated with increasing concentrations of wedelolactone (2.5, 5, and 10 μM), alongside mock and HBV-only controls. Our data revealed that wedelolactone dose-dependently reduced NF-κB p65 levels while restoring IκBα expression, suggesting inhibition of the classical inflammatory cascade (lanes 3 - 5). Concurrently, Nrf2 and its downstream effector HO-1 were markedly upregulated, while Keap1 was suppressed, indicating activation of the cellular antioxidant defense system (lanes 3 - 5). Real-time qPCR analysis of inflammatory cytokines TNF-α and IL-6 (Figure 6B) showed that HBV-only cells exhibited markedly elevated transcript levels compared with mock controls. TNF-α increased from 12.3 % in mock cells to 100.1 % in 1.3-mer HBV cells (8-fold increase, P < 0.001), while IL-6 rose from 15.5 % to 100.1 ± 5.8% (6.5-fold increase, P < 0.001). Wedelolactone treatment significantly and dose-dependently blunted these inflammatory responses. TNF-α mRNA levels fell to 76.7 %, 44.2 %, and 30.6 % at 2.5, 5, and 10 µM, corresponding to ~0.77-, 0.44-, and 0.31-fold of the HBV-only group (P < 0.05, P < 0.01, and P < 0.001, respectively, vs. HBV). IL-6 showed a similar pattern, decreasing to 65.4 %, 42.2 %, and 19.9 % (~0.65-, 0.42-, and 0.20-fold of HBV-only; P < 0.05, P < 0.01, and P < 0.001, respectively). Together, these data confirm that wedelolactone exerts a potent anti-inflammatory effect at the transcriptional level in HBV-infected hepatocytes. Consequently, qPCR analysis of antioxidant genes Nrf2, NQO1, and HO-1 (Figure 6C) was performed using the 1.3-mer HBV group as the calibrator (set to 100%). In HBV-only cells, basal expression was ~100% for all three genes. Wedelolactone treatment significantly altered this profile in a dose-dependent manner. Nrf2 mRNA levels decreased to 39 % and 48 % at 2.5 and 5 µM, respectively (0.4 - 0.5-fold vs. HBV, P < 0.01), but were modestly elevated to 108 % at 10 µM (1.1-fold, P < 0.05). In contrast, the downstream antioxidant enzymes were predominantly induced: NQO1 increased from 100 % in HBV-only cells to 58 %, 78 %, and 130 % at 2.5, 5, and 10 µM wedelolactone (0.6, 0.8, and 1.3-fold; P < 0.01, P < 0.01, and P < 0.05, respectively), while HO-1 rose from 100 % to 69 %, 78 %, and 128 % (0.7-, 0.8-, and 1.3-fold; P < 0.01, P < 0.01, and P < 0.05, respectively vs. HBV). Together, these data indicate that wedelolactone not only dampens HBV-induced inflammation but also enhances key components of the antioxidant defense system — particularly NQO1 and HO-1 — thereby contributing to a hepatoprotective, cytoprotective response in HBV-infected hepatocytes.

### 4.6. Schematic Model of Wedelolactone-Mediated Hepatoprotection in Hepatitis B Virus-Infected Hepatocytes

**Figure 7.**
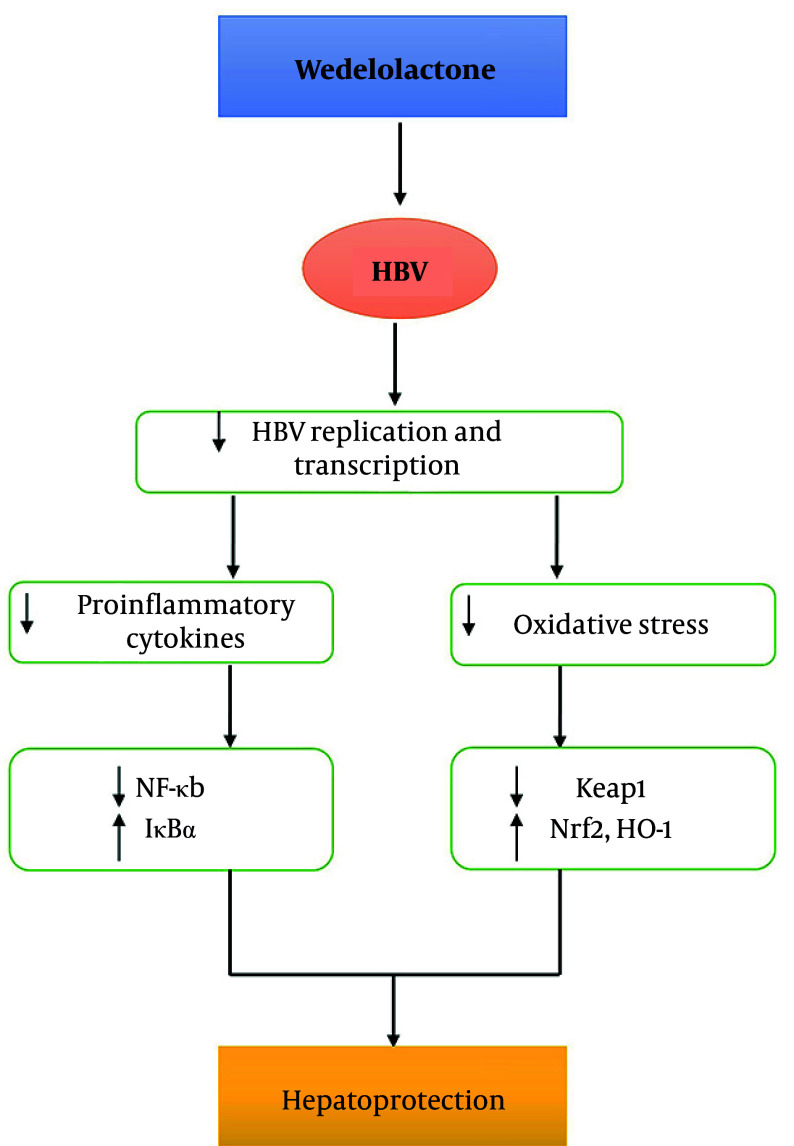
Schematic model of wedelolactone-mediated antiviral and hepatoprotective mechanisms in hepatitis B virus (HBV)-infected hepatocytes. Graphical summary illustrating the multi-pathway effects of wedelolactone on HBV replication, inflammatory cytokine production, NF-κB/IκB signaling, and antioxidant pathways (Nrf2/Keap1/HO-1 axis), culminating in hepatoprotection. The diagram was constructed based on experimental findings across Figure 1 and Figures 3-6.

This depicts the proposed mechanistic framework used by the current flowchart (Figure 7), where the use of the wedelolactone inhibitor led to a notable suppression of the viral outcome at the replication and transcription levels by reducing viral promoter activities, pgRNA/sRNA amounts, and DNA synthesis. There was also a notable reduction in the production of proinflammatory mediators (i.e., TNF-α and IL-6) and decreased oxidative stress through the upregulation of antioxidant mediators (Nrf2, HO-1) and the downregulation of the oxidative stress mediator (Keap-1). These combined effects contribute to the mediation of the hepatoprotective response, thus manifesting the dual role of wedelolactone in antiviral protective response and cellular cytoprotection through the intervention of the host's immune and redox responses. Apparently, this mechanistic basis presents the therapeutic potency of wedelolactone for the management of chronic liver injury resulting from HBV infection.

## 5. Discussion

Chronic HBV infection is not only a virological disorder but also a complex inflammatory and oxidative stress-driven disease that progressively damages the liver. While nucleos(t)ide analogues (NA) effectively suppress HBV replication, they do not eliminate covalently closed circular DNA (cccDNA) or reverse HBV-induced hepatic inflammation and oxidative injury, which are central to disease progression and hepatocarcinogenesis (17, 18). In parallel, population-level data continue to show that HBV/HCV remain substantial public-health burdens in high-risk settings, reinforcing the need for adjunct strategies that address both virological and host-injury components (19). In this context, multifunctional agents capable of simultaneously inhibiting viral replication and alleviating liver injury are of significant therapeutic value. Our study provides compelling evidence that wedelolactone, a natural coumestan derived from Eclipta prostrata, exhibits dual antiviral and hepatoprotective activity in HBV-infected hepatocytes. Wedelolactone treatment resulted in a concentration-dependent inhibition of HBV transcription, as demonstrated by suppression of Enh2/Core, Enh1/X, PreS1, and PreS2 promoter activities. This is a notable finding, as few natural compounds have been shown to directly modulate HBV promoter-driven transcription. This upstream regulatory effect translated into downstream suppression of pregenomic RNA (pgRNA), S RNA, and viral DNA synthesis, as well as reduced expression of HBcAg and HBsAg. These results suggest that wedelolactone exerts antiviral effects at both transcriptional and post-transcriptional levels, likely targeting host factors or transcriptional regulators exploited by HBV. Conceptually, diverse viruses across biological systems rely on host regulatory networks for transcription/translation and spread, and viral proteins can act as “rate-limiting” determinants of host range and tissue permissiveness (20, 21). Mechanistically, the broad, dose-dependent reduction across multiple HBV promoters suggests that wedelolactone may act upstream at the level of host transcriptional regulation rather than targeting a single promoter-specific element. HBV enhancer/promoter activity is strongly governed by hepatocyte-enriched transcription factors and nuclear receptors, including HNF4α and PPARα/RXRα complexes, which coordinate transcription from EnhII/Core and other regulatory regions (22, 23). Given wedelolactone’s reported ability to modulate inflammatory signaling (e.g., the NF-κB axis) and cellular stress responses, one plausible hypothesis is that it indirectly reduces the availability, DNA-binding competence, or co-activator recruitment of these hepatocyte transcriptional regulators, thereby diminishing HBV promoter-driven transcription. A second plausible hypothesis is epigenetic repression of viral transcriptional templates through altered co-regulator balance (e.g., reduced histone acetylation and/or increased recruitment of repressive chromatin modifiers), which could translate into decreased promoter output across multiple regulatory regions (24). These possibilities remain hypotheses in the absence of direct transcription factor occupancy or chromatin-state measurements; future studies will test them using ChIP-qPCR for HNF4α/PPARα binding and for activating/repressive histone marks on HBV templates, along with assessment of relevant co-activators/co-repressors and nuclear receptor signaling. Notably, virus–host regulation is frequently multi-layered and can involve coordinated control of transcriptional competence, RNA stability, and host defensive silencing — phenomena well documented in viral systems where coat proteins and movement proteins modulate permissiveness and infection outcomes (20, 21). In alignment with previous studies, wedelolactone also exhibited significant anti-inflammatory activity. HBV infection is known to induce persistent activation of NF-κB signaling, which contributes to chronic liver inflammation (25). Our data demonstrate that wedelolactone attenuates NF-κB p65 expression and restores IκBα levels, accompanied by a significant reduction in proinflammatory cytokines such as TNF-α and IL-6. These effects are consistent with earlier findings that wedelolactone inhibits IKK activity and prevents NF-κB nuclear translocation (25). Since inflammatory amplification is also a determinant of disease severity and biomarker patterns across viral infections, mechanistic alignment between antiviral control and reduced inflammatory injury is therapeutically meaningful (26). Additionally, wedelolactone increased Nrf2/HO-1/NQO1 expression and reduced Keap1 levels, which is consistent with engagement of the cellular antioxidant response (27). However, because intracellular ROS levels, Keap1–Nrf2 binding, and Nrf2 nuclear accumulation were not directly assessed in this study, the upstream mechanism(s) responsible for Nrf2 activation remain hypotheses. Future work will test whether wedelolactone (i) reduces oxidative burden and/or (ii) impairs Keap1-mediated sequestration of Nrf2 by performing ROS quantification assays (e.g., DCFDA), Nrf2 nuclear localization analyses (immunofluorescence and/or nuclear–cytoplasmic fractionation), and Keap1–Nrf2 interaction studies (co-immunoprecipitation). Importantly, wedelolactone was found to be non-cytotoxic at concentrations up to 10 µM and did not induce apoptosis in HBV-infected cells, as evidenced by preserved cell viability and lack of cleaved caspase-3 and PARP expression. This favorable safety profile, combined with its potent antiviral and immunomodulatory effects, positions wedelolactone as a promising candidate for further development (12). Beyond natural products, computational and structure-guided antiviral work against RNA viruses (including SARS-CoV-2) further supports the broader feasibility of host/virus-targeted multi-pronged strategies, although such approaches require careful validation in wet-lab systems (28). To our knowledge, this is the first study to comprehensively evaluate the impact of wedelolactone on HBV promoter activity, transcriptional output, DNA replication, viral antigen expression, and host response pathways within a single experimental framework. Importantly, when positioned against other extensively studied anti-HBV natural products, wedelolactone appears to occupy a distinct “dual-pathway” niche. Curcumin has been reported to suppress HBV, including effects linked to chromatin-level regulation such as reduced histone acetylation on cccDNA-associated templates, but its translational development is frequently constrained by formulation/bioavailability limitations and variability across experimental systems (29). Epigallocatechin gallate (EGCG) has shown anti-HBV activity in multiple reports and is often discussed in the context of pleiotropic host-targeted effects; however, published mechanisms are heterogeneous (and in some models more entry- or host-stress–biased rather than directly tied to multi-promoter transcriptional repression), which can complicate positioning as a defined transcriptional inhibitor (30, 31). Baicalin and related flavonoids are also repeatedly reported to dampen HBV markers and host inflammation, yet the mechanistic emphasis across studies often centers on broad immunomodulatory signaling rather than a clear, experimentally demonstrated upstream effect on multiple HBV promoter/enhancer elements (32). In contrast, the distinguishing feature in our dataset is the concentration-dependent suppression across several key HBV promoters (Enh2/Core, Enh1/X, PreS1, PreS2) accompanied by parallel attenuation of NF-κB–driven inflammatory outputs and activation of antioxidant-response markers, providing a coherent rationale for combined antiviral and hepatoprotective potential within a single experimental framework. It should be noted that the present study employs a 1.3-mer HBV transfection platform to interrogate HBV transcription/replication and host-response modulation under controlled conditions; therefore, our conclusions are confined to plasmid-driven HBV replication readouts and do not directly address viral entry or cccDNA establishment/maintenance. Validation in physiologically relevant infection systems (e.g., HepG2-NTCP, HepaRG, or primary human hepatocytes) will be an important next step to extend these findings across the complete HBV lifecycle. Complementary work in virology also highlights how assay design, primer selection, and detection strategies can influence interpretation and reproducibility, underscoring the need for robust validation across platforms (33, 34). Moreover, translational relevance is strengthened when mechanistic findings are evaluated alongside surveillance/epidemiologic signals in real-world viral disease contexts (35, 36). In conclusion, our findings suggest that wedelolactone offers a dual mode of action—direct inhibition of HBV transcription and replication, along with attenuation of HBV-induced inflammation and oxidative damage. These properties justify its further investigation as an adjunct or standalone candidate in chronic HBV therapy. Future studies should focus on validating these findings in vivo and exploring wedelolactone's effect on cccDNA transcription and host epigenetic regulation. Parallel advances in viral immuno-informatics and molecular vaccine design further emphasize the expanding toolkit available for virus-focused intervention development (37).

## Data Availability

The dataset presented in the study is available on request from the corresponding author during submission or after publication.
